# Mesenchymal Stromal/Stem Cells-Derived Exosomes as an Antimicrobial Weapon for Orodental Infections

**DOI:** 10.3389/fmicb.2021.795682

**Published:** 2022-01-04

**Authors:** Nazanin Jafari, Arezoo Khoradmehr, Reza Moghiminasr, Mina Seyed Habashi

**Affiliations:** ^1^Department of Endodontics, School of Dentistry, Bushehr University of Medical Sciences, Bushehr, Iran; ^2^The Persian Gulf Marine Biotechnology Research Center, The Persian Gulf Biomedical Sciences Research Institute, Bushehr University of Medical Sciences, Bushehr, Iran; ^3^Department of Stem Cells and Developmental Biology, Cell Science Research Center, Royan Institute for Stem Cell Biology and Technology, ACECR, Tehran, Iran

**Keywords:** exosomes, mesenchymal stromal/stem cells, dental infection controls, dentistry, orodental

## Abstract

The oral cavity as the second most various microbial community in the body contains a broad spectrum of microorganisms which are known as the oral microbiome. The oral microbiome includes different types of microbes such as bacteria, fungi, viruses, and protozoa. Numerous factors can affect the equilibrium of the oral microbiome community which can eventually lead to orodental infectious diseases. Periodontitis, dental caries, oral leukoplakia, oral squamous cell carcinoma are some multifactorial infectious diseases in the oral cavity. In defending against infection, the immune system has an essential role. Depending on the speed and specificity of the reaction, immunity is divided into two different types which are named the innate and the adaptive responses but also there is much interaction between them. In these responses, different types of immune cells are present and recent evidence demonstrates that these cell types both within the innate and adaptive immune systems are capable of secreting some extracellular vesicles named exosomes which are involved in the response to infection. Exosomes are 30–150 nm lipid bilayer vesicles that consist of variant molecules, including proteins, lipids, and genetic materials and they have been associated with cell-to-cell communications. However, some kinds of exosomes can be effective on the pathogenicity of various microorganisms and promoting infections, and some other ones have antimicrobial and anti-infective functions in microbial diseases. These discrepancies in performance are due to the origin of the exosome. Exosomes can modulate the innate and specific immune responses of host cells by participating in antigen presentation for activation of immune cells and stimulating the release of inflammatory factors and the expression of immune molecules. Also, mesenchymal stromal/stem cells (MSCs)-derived exosomes participate in immunomodulation by different mechanisms. Ease of expansion and immunotherapeutic capabilities of MSCs, develop their applications in hundreds of clinical trials. Recently, it has been shown that cell-free therapies, like exosome therapies, by having more advantages than previous treatment methods are emerging as a promising strategy for the treatment of several diseases, in particular inflammatory conditions. In orodental infectious disease, exosomes can also play an important role by modulating immunoinflammatory responses. Therefore, MSCs-derived exosomes may have potential therapeutic effects to be a choice for controlling and treatment of orodental infectious diseases.

## Introduction

The oral cavity is the second most diverse microbial community in the human body after the gut (Caselli et al., 2020). Numerous microorganisms including fungi, viruses, protozoa, and over 700 species of bacteria in this community are called “microbiome” (Deo and Deshmukh, 2019). The microbiome is a term that was coined by Joshua Lederberg, a Nobel Prize laureate, to explain the ecological community of symbiotic, commensal, and pathogenic microorganisms that share human body space (Kilian et al., 2016). Orodental infections are caused by changes in the balance of microbial populations or the dynamic relationship between them and the oral cavity (Cho and Blaser, 2012; Marsh et al., 2015). In addition, the oral cavity is exposed to external environmental microorganisms that can cause oral diseases (Gerba, 2015).

The host immune system plays an important role in defending against pathogens (Dunkelberger and Song, 2010). At first, It fights against pathogens through innate immunity and then through adaptive immunity (Cerny and Striz, 2019). Although the innate immune system response is general, non-specific, and does not directly target a single pathogen, it provides a defense barrier against all infectious agents (Aderem and Ulevitch, 2000). The skin and mucosal membranes act as a mechanical barrier against pathogens, also epithelial cells contain peptides that have antimicrobial properties (Ganz, 2003; Oppenheim et al., 2003). If the pathogens can get past the primary defense, the second line of defense becomes active (Frank, 2000). In the infected area, an inflammatory response begins due to stimulation of high blood pressure, the blood vessels dilate, and white blood cells leave the veins during diapause to fight the pathogen (Chen et al., 2018). The vessels diameter increase, because of the secretion of “histamine” from mast cells. Mast cells are a type of white blood cell and phagocytes that draw in pathogens and kill them. During the inflammatory response, the infected area becomes red, swollen, and painful (Janeway et al., 2001b; Csaba et al., 2003) and, the immune system may release substances that raise the body temperature and cause fever. An increase in temperature can decelerate the growth of pathogens and the immune system fights against infectious agents more quickly (Evans et al., 2015). Some phagocytic cells detect pathogenic cells and other kill cells in the body and digest them (Bain, 2017). In the human body, some proteins are normally inactive and activated in infection conditions. They create pores in the membrane of pathogenic cells and destroy them. These proteins are unable to distinguish different pathogens from each other and attack all pathogens non-specifically (Janeway et al., 2001a).

**FIGURE 1:**
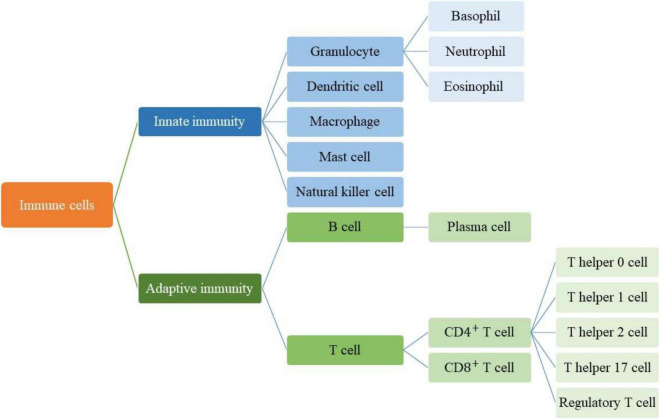
Immune system cells in innate and adaptive immunity responses.

Acquired or specific immunity is activated when a pathogen can cross the innate or non-specific immune mechanism (McDade et al., 2016). The cells of the body have signs that the immune system distinguishes them from other foreign cells (Rich and Chaplin, 2019). When the immune system encounters cells that do not have these signs, it recognizes them as aliens and attacks them through specific or acquired mechanisms, using lymphocytes and producing antibodies (Elgert, 2009). This mechanism develops during the growth of the human body. In this way, with the development of the human body and exposure to pathogens and various vaccinations, a library of antibodies from the cells of the immune system related to various pathogens is created in the body. This process is sometimes called “Immunological Memory” because immune cells remember their former enemies (Crotty and Ahmed, 2004). The acquired mechanism produces antibodies to protect the body against foreign agents, for example, if previous pathogens attack the body, it will produce antibodies more quickly and eliminate the infection (Jerne, 1973). Acquired immunity is caused by the presence of antigens. Antigens are usually located on the surface of pathogen cells, and each pathogen has its antigen (Lamm, 1997). The immune system responds to antigens by certain cells or by producing antibodies (Figure 1). Antibodies attack antigens and produce a signal that attracts phagocytes or other killer cells (Davies and Cohen, 1996). In the immune system, cells like mast cells (Raposo et al., 1997), epithelial cells (van Niel et al., 2001; Lin et al., 2005), antigen-presenting cells (Zitvogel et al., 1998), T lymphocytes (Anel et al., 2019), B lymphocytes (Kato et al., 2020), neutrophils (Vargas et al., 2016), and macrophage (Singhto et al., 2018) release small extracellular vesicles (EVs) which called “exosomes.”

## The Role of Exosomes in Microbial Infections

**FIGURE 2:**
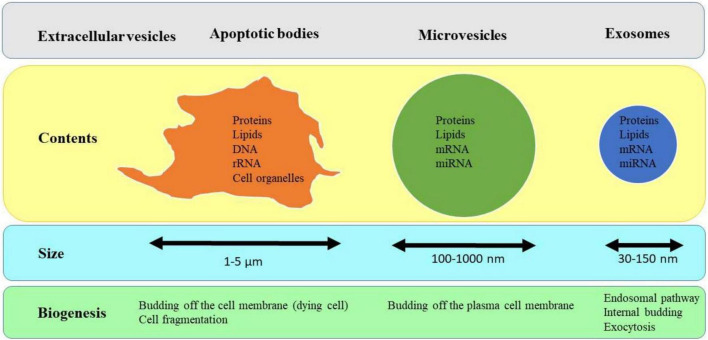
Three main subtypes of EVs and their properties.

EVs are made and secreted in normal and diseased states by most types of cells and have an essential role in intercellular communication and facilitate the immunity process They contain a wide range of lipid-bound nanoparticles that vary in size (Yanez-Mo et al., 2015; Maas et al., 2017; Herrmann et al., 2021). There is no certain agreement on markers or specific naming for EV subtypes, and EVs are usually classified according to their biogenesis pathway or their physical properties used for isolation (Théry et al., 2018). In fact, differences in size help to separate different types of EVs. Microvesicles, exosomes, and apoptotic bodies are the three main subtypes of EVs which are distinguished by their biogenesis, size, content, release pathways, and function (Figure 2; Karpman et al., 2017; Doyle and Wang, 2019; Ståhl et al., 2019).

In the late 1960s, for the first time, Bonucci (1967) and Anderson (1969) described small, secreted vesicles as small, 100-nm-diameter vesicles secreted by chondrocytes. A special subset of small EVs, between 30 and 150 nm in diameter, are known as exosomes that appear through endosomal biogenesis pathways (Willms et al., 2018; Tschuschke et al., 2020). A wide range of cell types can secrete exosomes, and the size of exosomes can vary even for exosomes secreted from a single cell line (Zhang et al., 2019). Exosomes consist of approximately 4,400 proteins, 194 lipids, 1,639 mRNAs, and 764 miRNAs and as secretory vesicles, the possibility of their physiological function has been defined (Mathivanan et al., 2012; Kim et al., 2013; Zhang et al., 2019; O’Brien et al., 2020). They can regulate the immune system and also interfere with biological processes. Pathogenic infections alter the number of exosomes, their contents, and membrane structure (Li et al., 2006; Zhang et al., 2018).

Infectious diseases like lower respiratory infections, malaria, diarrhea, tuberculosis (TB), human immunodeficiency virus (HIV) infection, and malaria are major reasons for morbidity and mortality worldwide and their treatment is challenging (Murray et al., 2014; Kirtane et al., 2021). Exosomes can interfere with the processes of infectious diseases. On the one hand, they can contribute to the pathogenesis of microorganisms, be effective in the progression of infection, and can fight against pathogens and infections. This functional variation of exosomes depends on the source of cells and their contents. To confirm this, Tables 1, 2 provide examples of the role of exosomes in infectious diseases. Briefly, Table 1 provides examples of the effects of exosomes on the pathogenicity of various microorganisms so that they cause and promote infections, and Table 2 lists several antimicrobial and anti-infective functions of exosomes in microbial diseases.

**TABLE 1:** The role of exosomes in the development of infections caused by various pathogens.

Microorganisms	Pathogens	Exosomes’ effects in promotion of infection	References
Bacteria	*Staphylococcus aureus*	*S. aureus*-derived exosomes spread the infection in the body by transmission of bacterial pore forming molecule α-toxin to distant cells.	Husmann et al., 2009
	*Bacillus anthracis*	Exosomes from *B. anthracis*-infected cells transport the lethal toxin virulence factor to sites distal to the infection.	Abrami et al., 2013
	*Helicobacter pilori*	Exosomes in *H. pylori* infection are secreted from cytotoxin-associated gene A (CagA)-expressing gastric epithelial cells enter the circulation and deliver CagA, a virulence factor, to distant organs and tissues.	Shimoda et al., 2016
Viruses	Human T-cell leukemia virus-1 (HTLV-1)	Exosomes produced by HTLV-1-infected T-cell lines deliver the viral transactivator (Tax) protein which can activate transcription in target cells.	Jaworski et al., 2014
	HIV-1	Exosomes derived from HIV-1-infected cells contain proteins of viral and cellular origin that inhibit target cell migration as well as dsRNA/ssRNA which can increase nuclear gene expression and promote infection.	Barclay et al., 2017
	Human herpesvirus 6 (HHV-6)	Exosomes derived from HHV-6-infected cells contain mature virions; therefore, they help spread infection more efficiently	Mori et al., 2008
	Hepatitis A virus (HAV)	Vacuolar protein sorting 4 homolog B (VPS4B) and ALG-2-interacting protein X (ALIX) play an important role in cloaking the HAV released from cells in host-derived membranes so protecting the virion from antibody-mediated neutralization. These enveloped viruses resemble exosomes and can escape the host immune system.	Feng et al., 2013
	Hepatitis B virus (HBV)	Exosomes derived from HBV-infected hepatocytes transport miR-21, miR-29a, and other miRs to Tamm-Horsfall Protein 1 (THP-1) macrophages, which results in suppressing Interleukin 12p35 (IL-12p35) mRNA expression and limitation of host innate immune response.	Kouwaki et al., 2016
	Hepatitis C virus (HCV)	*In vitro* study has shown that hepatic exosomes by protecting HCV against antibody neutralization can help transmit HCV infection.	Cosset and Dreux, 2014
	Hepatitis E virus (HEV)	HEV RNA-containing particles in an exosome fraction are infectious and cannot be neutralized by anti-HEV antibodies so they protect from the immune response.	Chapuy-Regaud et al., 2017
	Epstein-Barr virus (EBV)	EBV escapes immune responses by sequestering immune effectors like caspase-1, interleukin 1b (IL-1b), interleukin 18 (IL-18), and interleukin 33 (IL-33), in exosomes which are continuously secreted.	Ansari et al., 2013
	HIV type 1 (HIV-1)	Exosomes derived from HIV-1-infected cells allow HIV-1 to replicate inside resting human primary CD4 + T lymphocytes.	Arenaccio et al., 2014
Yeast	*Saccharomyces cerevisiae*	Cytosolic Sup35 NM prions are packaged into exosomes which are able to transmit the prion phenotype to neighboring cells.	Liu et al., 2016
Parasites	*Trypanosoma brucei*	*T. brucei rhodesiense* EVs mediating non-hereditary virulence factor transfer by containing the serum resistance-associated protein (SRA) and causing host erythrocyte remodeling, inducing anemia. Also, these EVs by transferring the SRA to *T. brucei* gain the ability to evade innate immunity.	Szempruch et al., 2016
	*Toxoplasma gondii*	Exosomes secreted by *T. gondii*-infected host cells. L6 cells could change the host cell proliferation and alter the host cell cycle and slight enhancement of S phase in L6 cells.	Kim et al., 2016
	*Trypanosoma cruzi*	*T. cruzi*-derived have been shown to increase the secretion of interleukin 4 (IL-4) and interleukin 10 (IL-10) and a diminished inducible nitric oxide synthase expression in CD4 + T cells and macrophages.	Trocoli Torrecilhas et al., 2009

**TABLE 2:** The function of different sources of exosomes in infectious disease.

Source of exosomes	Role of exosomes	References
Adipose tissue-derived MSCs	Combined with melatonin, an anti-inflammatory hormone, could limit inflammation caused by colitis *in vivo*.	Chang et al., 2019
Colonic lumen of IBD patients	Contribute to IBD diagnosis by containing significantly higher mRNA and protein levels of IL-6, IL-8, IL-10, and TNF-α compared with those from healthy individuals.	Larabi et al., 2020
Dendritic cells	Stimulate the responses of IL-4 and TNF-α and increase the IL-4 production in CD14 in *Malassezia sympodialis* infection.	Gehrmann et al., 2011
Dendritic cells	Stimulate the production of IgM, IgG3, and IgG1 types of anti-Cps14 responses in *Streptococcus pneumoniae* type 14 infection.	Colino and Snapper, 2007
Dendritic cells	Promote intestinal barrier function by activating NF-κB *via* the exosomal miR-146b in a murine model of colitis.	Nata et al., 2013; Alexander et al., 2015
HBV-infected hepatocytes	Stimulate MyD88, Toll-IL-1 receptor-containing adaptor molecule-1 (TICAM-1), and mitochondrial antiviral signaling (MAVS)-dependent pathways to induce NKG2D ligand expression and evoke NK cells.	Kouwaki et al., 2016
Healthy human semen	Prevent the spread of HIV-1 and reduce the intravaginal proliferation of AIDS in mice as well as the systematic spread of virus and viremia.	Madison et al., 2015
Human vaginal secretions	Have inhibitory properties against HIV-1 infection and protect women against HIV-1 infection as a female innate defense.	Smith and Daniel, 2016
Macrophages	Suppression of IFN-γ stimulated MHC class II and CD64 expression on BMMØ dependent on lipoproteins, TLR2 and MyD88 and also increase secretion of chemokines and stimulate migration of macrophages and splenocyte in *Mycobacterium tuberculosis* infection.	Singh et al., 2011, 2012
Macrophages	Induce Pro-inflammatory responses dependent on TLR 2, TLR4, and MyD88 in *Mycobacterium avium* infection.	Bhatnagar et al., 2007
MDSC	Reduce the severity of colitis by inhibiting Th1 proliferation and promoting Treg cell expansion.	Wang et al., 2016
MSCs	Inhibit inflammatory cytokine production by colonic macrophages stimulated with LPS and promote the polarization of these macrophages into M2 phenotype *in vitro* and also, alleviate colitis by inhibiting expression of IL-7 and iNOS in mouse colonic macrophages *in vivo*.	Mao et al., 2017; Cao et al., 2019
Mycoplasma-infected tumor cells	Activate the splenic B cells and increase the production of splenocytes cytokines.	Yang et al., 2012
**Plasmodium yoelii*-infected reticulocytes*	Decrease period of parasitemia and increase clearance of parasites, reticulocytosis, immune modulation, elicits IgG2a and IgG2b, and promoted survival time and protect mice from lethal infections.	Martin-Jaular et al., 2011
uMSCs	Contain some small RNAs (let-7f, miR-145, miR-199a, and miR-221) can prevent HCV replication by detecting specific cellular factors or binding directly to the virus genome and intercede the antiviral process.	Qian et al., 2016

*IBD, Inflammatory bowel disease; IgM, Immunoglobulin M; IgG3, Immunoglobulin G3; IgG1, Immunoglobulin G1; Cps14, capsular polysaccharide of S. pneumonia type 14; NF-κB, Nuclear factor- κB; MyD88, Myeloid differentiation primary response 88; NKG2D, Natural killer group 2 member D; NK cells, natural killer cells; AIDS, acquired immune deficiency syndrome; IFN-γ, Interferon gamma; BMMØ, bone marrow derived macrophage; TLR 2, toll like receptor 2; TLR 4, toll like receptor 4; MDSC, myeloid-derived suppressor cells; LPS, Lipopolysaccharides; IL-7, interleukin 7; iNOS, inducible nitric oxide synthase; IgG2a, Immunoglobulin G2a; IgG2b, Immunoglobulin G2b; uMSC, umbilical mesenchymal stem cells.*

## Orodental Infectious Disease

Orodental infectious diseases are caused by both pathogenic microorganisms and the loss of balance in the ecological community of symbiotic microorganisms in the oral cavity. Oral microbial diseases include a wide range of different diseases such as periodontitis and caries. If proper measures are not taken to control and treat mouth-infectious diseases, it can lead to whole-body systemic diseases (Table 3).

**TABLE 3:** Systemic diseases associated with oral microbiome and orodental infection.

The human body systems	Disease	References
Gastrointestinal system diseases	IBD	Read et al., 2021
	Gastrointestinal cancer risk increases	Meurman, 2010
	Pancreatic cancer	Fan et al., 2018
Nervous system diseases	Alzheimer’s disease	Miklossy, 1993; Riviere et al., 2002; Poole et al., 2013
Endocrine system diseases	Diabetes mellitus	Cianciola et al., 1982; Rylander et al., 1987; Emrich et al., 1991; Thorstensson and Hugoson, 1993; Casarin et al., 2013
	Adverse pregnancy outcomes (APOs)	Han et al., 2004, 2010; Madianos et al., 2013
	Obesity	Goodson et al., 2009
	Polycystic ovary syndrome (PCOS)	Lindheim et al., 2016
Human immune system diseases	Rheumatoid arthritis (RA)	Zhang et al., 2015
	HIV infection	Dang et al., 2012; Li et al., 2014a; Heron and Elahi, 2017
Cardiovascular system diseases	Atherosclerosis	Koren et al., 2011

### Periodontitis

The periodontium contains the supporting tissues around the structure of the teeth, such as the gingiva, cementum, junctional epithelium, periodontal ligament, and alveolar bone (Taba et al., 2005). Periodontal diseases are a result of periodontal structure destruction (Nanci and Bosshardt, 2006). The prevalence of periodontal disease is very high and more than 90% of adults worldwide suffer from it (Pihlstrom et al., 2005). There are two main categories of periodontal disease: gingivitis and periodontitis (Dorfer et al., 2004). Gingivitis is a milder form of periodontitis and is limited to gum tissue, but periodontitis occurs when the inflammation spreads to deeper tissues and causes loss of supporting connective tissue and alveolar bone (Kononen et al., 2019). The structure and texture of the periodontium can provide a suitable environment for the growth of various microorganisms (Cobb and Killoy, 1990). Microorganisms such as *Porphyromonas gingivalis*, *Tannerella forsythensis*, and *Treponema denticola* play an important role in the development of periodontal disease (Mineoka et al., 2008). *T. forsythensis*, *T. denticola*, and *Treponema lecithinolyticum* can be present in all phases of periodontal disease (Scapoli et al., 2015). *Porphyromonas endodontalis* and *p. gingivalis* are more specifically associated with periodontitis and *Capnocytophaga ochracea* and *Campylobacter rectus* associated with gingivitis (Scapoli et al., 2015).

### Dental Caries

Tooth decay is the most common chronic infectious disease which deals with the chronic and progressive destruction of hard tooth tissue (Ozdemir, 2013; Rathee and Sapra, 2020). In this disease, the hard tooth tissue (enamel and dentin) loses calcium and phosphorus minerals due to acid secretion from cariogenic bacteria (mainly *Streptococcus mutans*) (Moynihan and Petersen, 2004; Selwitz et al., 2007; Krzysciak et al., 2014). There are various causes for caries, but in general, the four main factors of tooth-adherent specific bacteria, time, susceptible tooth surface, and fermentable carbohydrates play a role in tooth decay (Tahir and Nazir, 2018). These four factors always cause caries, and if each one is not present, the tooth will not decay (Fejerskov, 1997; Sheiham, 2001; Wade, 2013; Kidd and Fejerskov, 2016; Tahir and Nazir, 2018). Tooth decay, in addition to its high prevalence, affects a wide range of age groups, and from children to the elderly, they are at risk for tooth decay (Smith and Szuster, 2000). The most harmful type of caries occurs in childhood and is named “early childhood caries” which has become a common public health problem among preschool children worldwide (Colak et al., 2013; Alazmah, 2017). Numerous factors, including the oral microbiome, affect the incidence of tooth decay in children (Dzidic et al., 2018). Bacteria are considered the main pathogen in tooth decay (Dzidic et al., 2018). Different lactobacilli promote the development of dental caries, but the most important microorganism in the development of dental caries is *S. mutans* (Loesche, 1996).

### Oral Leukoplakia

In 1877, oral leukoplakia was described for the first time by Schwimmer (1877) Oral leukoplakia is one of the most common diseases of the oral mucosa which has malignant potential (van der Waal et al., 1997). According to the Pindborg study, leukoplakia is a white patch on the oral mucosa that cannot be removed and there is no other clinical diagnosis (Mehta et al., 1969; Bánóaczy, 1983). Different microorganisms like *Fusobacterium*, *Leptotrichia*, *Campylobacter*, and *Rothia* species were detected in oral leukoplakia (Amer et al., 2017).

### Oral Squamous Cell Carcinoma

Oral squamous cell carcinoma is the eighth most common cancer worldwide and is the most common oral malignancy (Scully and Bagan, 2009). Numerous hypotheses have been proposed for the association of microorganisms and their products with oral cancer (Perera et al., 2016). Acetaldehyde converted from ethanol, reactive oxygen species, reactive nitrogen species, and volatile sulfur compounds by bacteria are some examples of carcinogenic substances which can cause oral cancer (Meurman and Uittamo, 2008). The metabolization of alcohol to acetaldehyde can be happened by *Streptococcus gordonii*, *Streptococcus mitis*, *Streptococcus oralis*, *Streptococcus salivarius*, *Streptococcus sanguinis*, and Candida by the using of alcohol dehydrogenase enzyme (Mantzourani et al., 2009; Marttila et al., 2013). Also, hydrogen sulfide (H2S), methyl mercaptan (CH3SH), and dimethyl sulfide [(CH3)2S] are produced by *P. gingivalis*, *Prevotella intermedia*, *Aggregatibacter actinomycetemcomitans*, and *Fusobacterium nucleatum* (Nakamura et al., 2018; Suzuki et al., 2019).

## Application of Stem Cells-Derived Exosomes in Orodental Infections

Mesenchymal stromal/stem cells (MSCs) are adult pluripotent stem cells with self−renewing potential that have been administered in different types of diseases (Undale et al., 2009; Fitzsimmons et al., 2018). The unique biomedical characteristic of MSCs is their stemness by stimulating their proliferation and differentiating into multi-lineage cells (da Silva Meirelles et al., 2006). MSCs are immunologically safe. Low expression of major histocompatibility complex (MHC) class I molecules and expression of only a few MHC class II molecules make MSCs low immunogenicity cells (Hass et al., 2011; Lee et al., 2014). Immunomodulatory and regenerative functions of MSCs have been shown in various types of diseases (Zappia et al., 2005; Corcione et al., 2006; Wang et al., 2013; Forbes et al., 2014; Le Blanc and Davies, 2015). MSCs-derived exosomes also have angiogenic potential that can improve ischemic diseases (Babaei and Rezaie, 2021). Senescence of MSCs during *in vitro* expansion makes the cells less productive and can increase disease severity by causing inflammaging (Lee and Yu, 2020). Also, weak engraftment of infused MSCs, and donor-dependent variations are some limitations of application MSCs in clinical trials (Karp and Leng Teo, 2009; Siegel et al., 2013; Li et al., 2016). An alternative method to improve MSC-based therapy is to use exosomes (Zavatti et al., 2020). Being free of immunogenic problems and not being trapped in the lung or liver like infused MSCs, and keeping the therapeutic functions of their cells of origin make MSC exosomes more suitable for clinical application than MSCs (Table 4; U.S. National Library of Medicine clinicaltrials.gov, 2021). The immunomodulatory function of MSCs and MSC-derived exosomes is the most important clinical feature of their application (Kang et al., 2020). Recent studies show that MSCs can inhibit T cells, B cells, natural killer cells, and dendritic cells and result in immune suppression (Bocelli-Tyndall et al., 2007; Li et al., 2012). Regarding MSCs properties, they have been used in clinical trials over several decades (Kabat et al., 2020). The MSCs mainly modulate the activity of the immune system by paracrine agents and exosomes, and the exosomes play an important role in cellular communication (Xu et al., 2016). MSCs-derived exosomes have a role in tissue regeneration, infection treatment, and inflammation control (Afshar et al., 2021; Zhankina et al., 2021).

**TABLE 4:** Some applications of MSCs-derived exosomes in recent clinical trials (U.S. National Library of Medicine clinicaltrials.gov, 2021).

Disease type	Official study title	Condition or disease	Intervention/treatment	Last update	ClinicalTrials.gov Identifier
Cancer	Phase I study of mesenchymal stromal cells-derived exosomes with KrasG12D siRNA for metastatic pancreas cancer patients harboring KrasG12D mutation	KRAS NP_004976.2:p.G12D Metastatic pancreatic adenocarcinoma Pancreatic ductal adenocarcinoma Stage IV pancreatic cancer AJCC v8	Mesenchymal stromal cells-derived exosomes with KRAS G12D siRNA	April 29, 2021	NCT03608631
Cardiovascular diseases	Safety and efficacy of allogenic mesenchymal stem cells derived exosome on disability of patients with acute ischemic stroke: a randomized, Single-blind, Placebo-controlled, Phase 1, 2 trial	Cerebrovascular disorders	Exosome	January 25, 2021	NCT03384433
COVID-19 treatment	A Pilot clinical study on aerosol inhalation of the exosomes derived from allogenic adipose mesenchymal stem cells in the treatment of severe patients with novel coronavirus pneumonia	Coronavirus	MSCs-derived exosomes	September 7, 2020	NCT04276987
	A tolerance clinical study On aerosol inhalation of mesenchymal stem cells exosomes in healthy volunteers	Healthy	Biological: 1X level of MSCs-Exo Biological: 2X level of MSCs-Exo Biological: 4X level of MSCs-Exo Biological: 6X level of MSCs-Exo Biological: 8X level of MSCs-Exo	August 4, 2021	NCT04313647
	A phase I/II randomized, double blinded, placebo trial to evaluate the safety and potential efficacy of intravenous infusion of zofin for the treatment of moderate to SARS related to COVID-19 infection vs. placebo	Corona virus infection COVID-19 SARS Acute respiratory distress syndrome	Biological: Zofin Other: Placebo	February 23, 2021	NCT04384445
	Bone marrow mesenchymal stem cell derived extracellular vesicles infusion treatment for COVID-19 associated acute respiratory distress syndrome (ARDS): A phase II clinical trial	COVID-19 ARDS Pneumonia, Viral	Biological: DB-001 Other: Intravenous normal saline	July 14, 2021	NCT04493242
	Mesenchymal stem cell exosomes for the treatment of COVID-19 positive patients with acute respiratory distress syndrome and/or novel coronavirus pneumonia	COVID-19 Novel coronavirus pneumonia Acute respiratory distress syndrome	MSC-exosomes delivered intravenously every other day on an escalating dose: (2:4:8) MSC-exosomes delivered intravenously every other day on an escalating dose (8:4:8) MSC-exosomes delivered intravenously every other day (8:8:8)	July 21, 2021	NCT04798716
	The protocol of evaluation of safety and efficiency of method of exosome inhalation in SARS-CoV-2 associated two-sided pneumonia	COVID-19 SARS-CoV-2 pneumonia COVID-19	EXO 1 inhalation EXO 2 inhalation Placebo inhalation	November 4, 2020	NCT04491240
	The extended protocol of evaluation of safety and efficiency of method of exosome inhalation in COVID-19 associated two-sided pneumonia	COVID-19 SARS-CoV-2 pneumonia COVID-19	EXO 1 inhalation EXO 2 inhalation Placebo inhalation	October 26, 2020	NCT04602442
Immune diseases	Phase 1 study of the effect of cell-free cord blood derived microvesicles On β-cell mass in type 1 diabetes mellitus (T1DM) patients	Diabetes mellitus type 1	MSC exosomes	May 14, 2014	NCT02138331
	Effect of umbilical mesenchymal stem cells derived exosomes on dry eye in patients with chronic graft vs. host diseases	Dry eye	Umbilical mesenchymal stem cells derived exosomes	February 21, 2020	NCT04213248
	Effect of adipose derived stem cells exosomes as an adjunctive therapy to scaling and root planning in the treatment of periodontitis: A human clinical trial	Periodontitis	Adipose derived stem cells exosomes	February 17, 2020	NCT04270006
	Exosome of mesenchymal stem cells for multiple organ dysfuntion syndrome after surgical repaire of acute type A aortic dissection: a Pilot Study	Multiple organ failure	MSC exosomes	May 6, 2020	NCT04356300
Neurological diseases	Focused ultrasound delivery of exosomes for treatment of refractory depression, Anxiety, and Neurodegenerative dementias	Refractory depression anxiety, Disorders neurodegenerative diseases	Exosomes	March 5, 2021	NCT04202770
	The use of exosomes In craniofacial neuralgia	Neuralgia	Exosomes	March 5, 2021	NCT04202783
	Open-label, Single-center, Phase I/II clinical trial to evaluate the safety and the efficacy of exosomes derived from allogenic adipose mesenchymal stem cells in patients with mild to moderate dementia Due to Alzheimer’s disease	Alzheimer’s disease	Biological: Low dosage MSCs-Exos administrated for nasal drip Biological: Mild dosage MSCs-Exos administrated for nasal drip Biological: high dosage MSCs-Exos administrated for nasal drip	June 25, 2021	NCT04388982
Wound healing	Mesenchymal stem cells derived exosomes promote healing of large and refractory macular holes	Macular holes	Exosomes derived from mesenchymal stem cells (MSC-Exo)	April 6, 2021	NCT03437759
	A safety study of the administration of mesenchymal stem cell extracellular vesicles in the treatment of dystrophic epidermolysis bullosa wounds	Dystrophic epidermolysis bullosa	AGLE 102	June 24, 2021	NCT04173650

Periodontitis is an inflammatory and destructive disease that has a relationship with several factors such as the pathogens, host inflammation, and immune responses, and the imbalance of multiple T helper cells 17 (Th17)/regulatory T cell (Treg) related cytokines (Wang et al., 2014; Silva et al., 2015; Pan et al., 2019). Bacterial infection is a primary factor in the development of periodontitis, but what ultimately causes periodontitis is improper regulation of the host immune system and inflammatory response (Hajishengallis, 2014, 2015). Th17 cells play a destructive role in the immune balance of periodontitis (Zhao et al., 2011). Over-regulation of Th17 and improper regulation of Treg may lead to periodontal disease through immune-mediated tissue destruction (Zhao et al., 2011; Yang et al., 2014; Karthikeyan et al., 2015). Periodontal ligament stem cells (PDLSCs)-derived exosomes have a similar role with exosomes from MSCs and PDLSCs-derived exosomes contain microRNA−155−5p and regulate Th17/Treg balance by targeting sirtuin−1 in chronic periodontitis (Zheng et al., 2019).

Interleukin-1 (IL-1) and tumor necrosis factor alpha (TNF-α) are pro-inflammatory cytokines that are needed for periodontal inflammation and alveolar bone resorption (Delima et al., 2001; Grauballe et al., 2015). Macrophages that are activated by bacteria can release many inflammatory cytokines, causing gingiva destruction and alveolar bone resorption (Spiller and Koh, 2017; Dutzan et al., 2018; Garaicoa-Pazmino et al., 2019). Macrophages can be divided into two groups which are known as pro-inflammatory macrophages and anti-inflammatory macrophages and periodontal destruction occur following the imbalance of pro-inflammatory/anti-inflammatory macrophages (Gonzalez et al., 2015; Wynn and Vannella, 2016; Zhuang et al., 2019). Pro-inflammatory macrophages play an important role in the production of many inflammatory cytokines such as interleukin 1 beta (IL-1β) and TNF-α. Also, they can stimulate T cells and neutrophils, which cause the destruction of alveolar bone, and they can increase the local expression of receptor activator of nuclear factor ligand (RANKL), which causes osteoclast differentiation in the periodontium (Darveau, 2010; Hienz et al., 2015). In contrast, anti-inflammatory macrophages by secreting the anti-inflammatory mediators play a significant role in the elimination of inflammation and tissue regeneration and contribute to efferocytosis of the apoptotic osteoblastic cells so that mediating bone formation (Zhang et al., 2012; Shapouri-Moghaddam et al., 2018).

Dental pulp stem cells (DPSCs) as a population of dental−derived mesenchymal stem cells have easy accessibility and minimal ethical concerns for use (Mahdiyar et al., 2014; Potdar and Jethmalani, 2015; Mehrabani et al., 2017). The DPSCs have beneficial immunomodulatory and anti-inflammatory properties and have a regulating effect on macrophages of the immune system (Lee et al., 2016; Omi et al., 2016; Galipeau and Sensebe, 2018). Since the therapeutic effects of stem cells are mainly related to the release of paracrine agents, stem cell-derived exosomes, as one of the most important paracrine mediators, show therapeutic effects through immunomodulation (Sun et al., 2018; Riazifar et al., 2019). DPSC-derived exosomes containing miR-1246 can facilitate the conversion of pro-inflammatory macrophages to anti-inflammatory macrophages in the periodontium of mice with periodontitis and accelerate the healing of alveolar bone and the periodontal epithelium (Shen et al., 2020).

In connection with the issue of infectious diseases, exosomes, in addition to treatment, can also help in the diagnosis of infectious diseases. For instance, hand, foot, and mouth disease (HFMD) is a common acute viral infection that has spread worldwide (Guerra et al., 2017). Human enterovirus 71 (EV71) and coxsackie virus A16 (CVA16) are the two main causes of HFMD (Yan et al., 2001; Osterback et al., 2009). HFMD has mild and severe forms which are known as mild HFMD and extremely severe HFMD (Jia et al., 2014), EV71 can cause extremely severe HFMD in which severe neurological symptoms occur and significant mortality (Huang et al., 1999). Many children with extremely severe HFMD die before a definitive diagnosis. There are no effective and reliable methods and tools for diagnosing (Li et al., 2014b; Hossain Khan et al., 2018). A study has shown that patients with different HFMD conditions express a specific type of exosomal miRNA profile (Jia et al., 2014). In fact, these exosomes provide a supplemental biomarker for differential infection stage at an early stage. Therefore, by examining the exosomal content, the disease can be diagnosed, and its different forms can be distinguished from each other (Jia et al., 2014). The immunomodulatory properties of exosomes have enhanced their use in the field of cancer biology. For example, dendritic cells-derived exosomes called “Dexosomes” can be used as a cell-free vaccine for cancer immunotherapy (Nikfarjam et al., 2020). Also, homeostasis and metastasis of tumor cells can change by exosomal and autophagy pathways (Salimi et al., 2020). Radiotherapy may affect the mechanism of paracrine intercellular communication within irradiated tumor tissue and surrounding cells (Jabbari et al., 2019).

## Future Perspective of Exosome Therapy

Over the last decades, the knowledge about biogenesis, molecular content, and biological function of exosomes have significant progress and a considerable amount of manuscripts have been published in this field. Exosome therapy as a cell-free therapy is emerging as a promising strategy for the treatment of several diseases, in particular inflammatory conditions. The characteristic properties of exosomes, including the transmission of exosomal competent, protecting it from extracellular degradation, and delivering it in a highly selective manner to target cells, have led to their numerous uses in various fields of treatment. The use of exosomes in clinical applications as well as in the treatment of diseases has both advantages and challenges, some of which are listed in Table 5. Despite the existing limitations, the use of exosomes as a new method in various fields of medical science is phenomenal and inspiring that need more data collection.

**TABLE 5:** Advantages and limitations of exosomes therapy in clinical applications (Tian et al., 2010; Takahashi et al., 2013; Lötvall et al., 2014; Yu et al., 2014; Théry et al., 2018; Xing et al., 2020; Babaei and Rezaie, 2021).

Advantages	Limitations
Efficient cellular entry	Controversies in defining exosome dosage
Excellent immune-compatibility	Difficulty in identification of isolation and purification strategy in order to produce optimal results
Exerting different therapeutic mechanisms simultaneously	Lack of reliable methods for distinguishing them from other EVs
Free of ethical issues	Lack of standardized methods for large-scale production
Good stability and protection by having bilayer lipid membrane	Needing appropriate, safe, and confident cell sources of exosomes based on their intended therapeutic use
High diagnostic sensitivity and specificity by having multiple diagnostic parameters	Needing considerable attention of stability and storage strategies for clinical and commercial success as off-the-shelf diagnostic and therapeutic tools
Intrinsic ability to traverse biological barriers	Short half-life and quick clearance
Lower toxicity	Uptake capacity of target cells
Minimal trauma than other diagnostic methods in diagnosis of disease	
Modification ability	
Not immunogenic	
Potential targeting ability by the surface-specific domain	
Safe and non-tumorigenic	
Wide availability in various bodily fluids	

## Conclusion

The oral cavity as a part of the digestive system which is in close contact with the external environment of the body and also by having its special microbiome is prone to a wide range of infectious diseases. In infectious diseases, the pathogenic mechanism of the microorganism is significantly affected by a special type of EVs called exosomes. In this way, these exosomes can be effective in the process of disease development and progression, as well as in the face of preventing and limiting the disease. Exosomes also play an important role in microbial infections by regulating the host immune system. In addition, exosomes can be used in the diagnosis of infectious diseases. Due to the importance of treating oral infectious diseases as well as the ease of using non-cellular therapies, mesenchymal stromal/stem cells-derived exosomes can be considered as a suitable and available option for the treatment of orodental infectious diseases that require more and more extensive studies in the future.

## Author Contributions

NJ wrote the manuscript with support from AK and RM. MS helped supervise the project. All authors reviewed the manuscript and approved the final version of the manuscript.

## Conflict of Interest

The authors declare that the research was conducted in the absence of any commercial or financial relationships that could be construed as a potential conflict of interest.

## Publisher’s Note

All claims expressed in this article are solely those of the authors and do not necessarily represent those of their affiliated organizations, or those of the publisher, the editors and the reviewers. Any product that may be evaluated in this article, or claim that may be made by its manufacturer, is not guaranteed or endorsed by the publisher.
